# Rural–Urban and Appalachian Disparities in Geographic Access to Colonoscopy and Screening Prevalence Across the Contiguous US

**DOI:** 10.5888/pcd23.260126

**Published:** 2026-09-17

**Authors:** Sima Namin, R. Eric Heidel, Martin Whiteside, Jennifer Ferris, Jonathan S. Wall, James M. McLoughlin

**Affiliations:** 1 Office of Research Support, University of Tennessee Health Science Center College of Medicine–Knoxville, Knoxville, Tennessee; 2 Department of Surgery, University of Tennessee Health Science Center College of Medicine–Knoxville, Knoxville, Tennessee; 3 Office of Cancer Surveillance, Tennessee Department of Health, Nashville, Tennessee; 4 Department of Medicine, University of Tennessee Health Science Center College of Medicine–Knoxville, Knoxville, Tennessee

## Abstract

**Introduction:**

Colorectal cancer (CRC) incidence and mortality rates are disproportionately high in rural and Appalachian communities, where geographic barriers and specialist shortages may limit access to colonoscopy.

**Methods:**

We conducted a national cross-sectional geospatial analysis of all contiguous US census tracts. We identified colonoscopy providers by using National Provider Identifier specialty designations and colonoscopy procedure indicators. Geographic access was quantified by using a 4-band enhanced 2-step floating catchment area (E2SFCA) method. We used small-area estimation for colonoscopy from Centers for Disease Control and Prevention PLACES to determine CRC screening prevalence. We compared E2SFCA distributions across rural–urban and Appalachian–non-Appalachian using the Kruskal–Wallis test with Benjamini–Hochberg-adjusted Dunn post hoc pairwise comparisons; we modeled screening prevalence using a mixed-effects beta regression analysis.

**Results:**

Urban census tracts tended to rely on delivery of colonoscopies by gastroenterologists and ambulatory surgical centers, whereas rural census tracts relied more on general surgeons, particularly for screening (vs diagnostic/therapeutic colonoscopy). Geographic access to colonoscopy providers varied little across census tracts, with only modest differences by rurality and Appalachian designation. In mixed-effects beta regression, colonoscopy access was not significantly associated with screening prevalence while both rural and Appalachian census tracts had slightly lower screening prevalence compared with their counterparts (rural vs urban: Exp(β) = 0.994; *P* = .02; Appalachian vs non-Appalachian: Exp(β) = 0.976; *P* < .001).

**Conclusion:**

Better geographic access does not translate into meaningfully higher screening prevalence. Structural, socioeconomic, and referral-related barriers likely attenuate the effect of geographic proximity on screening behavior. Further integration of claims-based utilization and patient-level factors is needed to clarify pathways between access and CRC screening.

**Summary**

**What is already known on this topic?**

Rural and Appalachian communities have a higher incidence of colorectal cancer and a lower prevalence of screening; distance and specialist shortages may limit access to colonoscopy.

**What is added by this report?**

We found only modest differences in geographic access to colonoscopy providers by rurality or Appalachian designation and no significant association between access and screening prevalence. Rural areas relied more on general surgeons as compared with urban areas; rural areas also had fewer ambulatory surgical centers.

**What are the implications for public health practice?**

Planning should go beyond siting endoscopy services to address referral pathways, capacity, affordability, and patient barriers and should support rural workforce models; integrating claims and utilization data can aid in targeting interventions.

## Introduction

Colorectal cancer (CRC) is the fourth most common type of cancer, representing 7.6% of all new cancer cases in the US (1). Yet it is also among the cancers that are most preventable through guideline-recommended screening (2). Colonoscopy, widely used for both screening and diagnostic follow-up, requires specialized facilities, trained health care providers, and adequate geographic access to colonoscopy providers, all of which are known to vary widely across regions (3). Disparities in geographic access to colonoscopy providers are especially pronounced in central and southern Appalachia, where CRC incidence and mortality are among the highest in the nation. In 2022, Mississippi, Kentucky, and West Virginia had some of the highest incidence rates (43.7, 46.1, and 44.3 per 100,000 persons, respectively), coupled with elevated CRC mortality rates in 2023 (17.7, 17.5, and 16.5 per 100,000 persons, respectively) (4). Rural populations and residents of the Appalachian region in particular have elevated CRC incidence and mortality rates, lower screening uptake, and reduced access to specialty care (5).

These disparities suggest that geographic access to colonoscopy providers may contribute to observed disparities in screening uptake and ultimately to disparities in CRC outcomes. Previous studies highlighted that spatial access is only 1 dimension that may influence screening behavior (6). Evidence evaluating whether geographic access to colonoscopy providers is associated with community-level screening uptake across rural/urban and Appalachian contexts remains limited.

This study aimed to 1) quantify geographic access to colonoscopy providers for all contiguous US census tracts using the enhanced 2-step floating catchment area (E2SFCA) method (7); 2) compare access across rural and urban census tracts and Appalachian and non-Appalachian regions; and 3) assess whether geographic access is associated with CRC screening prevalence. By integrating national provider data, travel times, and census tract–level screening estimates, this study aimed to provide an evaluation of geographic access to colonoscopy providers because such access may contribute to disparities in CRC screening prevalence.

## Methods

We conducted a national cross-sectional geospatial analysis to evaluate geographic access to colonoscopy providers and the association of geographic access with CRC screening prevalence across all contiguous US census tracts. We applied the E2SFCA method to quantify travel-time–weighted access to colonoscopy-performing clinicians and facilities, and we examined differences by rurality and Appalachian designation.

### Data sources

#### Location of colonoscopy providers

We identified colonoscopy-performing providers using the 2023 Medicare Physician and Other Practitioners by Provider and Service Public Use File (PUF) (8), which contains procedure-level claims aggregated by National Provider Identifier. We classified providers as performing colonoscopy if they billed at least 1 colonoscopy-related Current Procedural Terminology (CPT)/Healthcare Common Procedure Coding System (HCPCS) code. Colonoscopy procedures were defined using CPT/HCPCS codes, including screening codes (G0105, G0121) and diagnostic and therapeutic colonoscopy codes (45378–45398), consistent with Centers for Medicare & Medicaid Services (CMS) and CPT coding definitions. We calculated total colonoscopy volume as the sum of services across these codes for each provider–address–setting record. We identified providers based solely on claims evidence of colonoscopy performance using CPT/HCPCS codes; specialty designation was not used to determine inclusion but was used for subsequent classification.

Using National Provider Identifiers, we linked providers to the CMS National Plan and Provider Enumeration System (NPPES) May 2025 Data Dissemination File (9) and obtained practice-location addresses and taxonomy-based specialty classification. To determine provider specialty, we used NPPES taxonomy codes recorded across up to 15 taxonomy fields per provider. We prioritized the primary taxonomy designation when it was available; otherwise, we used the first nonmissing taxonomy code and retained all available taxonomy codes for classification. To classify providers, we used the following taxonomy codes: 207RG0100X (gastroenterology), 208C00000X (colon and rectal surgery), 208600000X (general surgery), 207RI0011X (internal medicine–gastroenterology), 207R00000X (internal medicine), 207Q00000X (family medicine), 363L00000X (nurse practitioner), 363A00000X (physician assistant), 282N00000X (hospital organization), and 261QA1903X (ambulatory surgical center), with remaining providers categorized as “other.”

No minimum service threshold was applied beyond the presence of at least 1 qualifying colonoscopy claim. We derived practice-location addresses from the Medicare PUF when available, with NPPES practice-location fields used as fallback. We used place-of-service codes to classify the care setting (eg, office, hospital outpatient, ambulatory surgical center). To create an inventory of colonoscopy provider sites and conduct site-level analyses, we combined and deduplicated provider records across office, hospital outpatient, and ambulatory surgical center settings to unique site locations based on geocoded coordinates.

### Population data

We used 2020 US Census tract boundaries (TIGER/Line) (10), merged with American Community Survey 2019–2023 5-year estimates (11). We defined demand for colonoscopy screening at the census tract level as the population aged 45 to 74 years. We extracted census tract–level counts for all study census tracts and used them as the demand input in accessibility modeling. To improve spatial representation of population demand, we used block group–level American Community Survey 2019–2023 estimates of the population aged 45 to 74 years to compute population-weighted census tract centroids, which served as origins in the accessibility analysis.

#### CRC screening prevalence

We obtained data on census tract–level CRC screening prevalence from the PLACES dataset (2023 release) (12), which provides small-area modeled estimates of the percentage of adults aged 45 years or older who meet guideline-recommended CRC screening. We joined these estimates to census tracts using standard geographic identifiers.

#### Rurality and Appalachian designation

We assigned Rural–Urban Commuting Area (RUCA) codes to assign rurality to census tracts (13), dichotomized as urban (codes 1–3) or rural (4–10). Appalachian designation was assigned using the Appalachian Regional Commission county-level designation (14) and mapped to census tracts. We used classifications to stratify results and test for effect modification in rural–Appalachian vs nonrural–non-Appalachian areas.

#### Travel time data

We computed a national origin–destination travel–time matrix between every census tract centroid and every colonoscopy provider. We generated travel times using the Open-Source Routing Machine engine applied to a national OpenStreetMap network (OpenStreetMap Foundation, https://osmfoundation.org).

### E2SFCA method

We employed an E2SFCA method that incorporates both service capacity and travel-time–based distance decay. Using a national origin–destination travel–time matrix linking census tracts to colonoscopy service provider locations, we retained census tract–colonoscopy service provider site pairs with driving times of 60 minutes or less. We applied a 4-band stepwise distance decay function with the following weights: 0 to 15 minutes (weight = 1.00), 15 to 30 minutes (0.75), 30 to 45 minutes (0.50), and 45 to 60 minutes (0.25). We excluded origin–destination pairs beyond 60 minutes, corresponding to a weight of zero outside the catchment.

Prior studies showed that accessibility patterns can be sensitive to both the number of distance bands and the choice of decay coefficients (15,16). To address this, we implemented a multiband distance decay structure and conducted sensitivity analyses using alternative weighting schemes.

#### Step 1: Provider-to-population ratios

For each colonoscopy provider site *j*, we identified all census tracts* i *within a 60-minute travel-time catchment. Demand for census tract *i* was defined as the American Community Survey 2019–2023 population aged 45 to 74 years. A weighted population load was computed using the decay weights *W_ij_.* The provider-to-population ratio was calculated as

$R_{j}=\frac{S_{j}}{\sum_{i\in \{dij\leq 60\}}Pi\;Wij}$

Where *S_j_* represents the number of colonoscopy providers at location *j*, and *P_i_* is census tract–level demand.

#### Step 2: Tract-level accessibility score

For each census tract *i* we identified all colonoscopy provider sites *j *reachable within 60 minutes. Accessibility was computed as the weighted sum of provider ratios:

$A_{i}=\sum_{j\in \{dij\leq 60\}}Rj\;Wij$

This resulted in a continuous accessibility index reflecting both supply and travel time. Census tracts with no providers within 60 minutes received an accessibility score of zero under this access definition.

### Statistical analysis

We examined differences in colonoscopy provider mix by rurality, Appalachian designation, and procedure type. Provider mix was defined as the proportion of total colonoscopy services attributable to each provider category (gastroenterology, general surgery, ambulatory surgical centers, and other) within each census tract. We classified colonoscopy claims billing provider specialty, gastroenterology, general surgery, and ambulatory surgical centers, and by procedure indication (screening vs diagnostic/therapeutic). For each census tract, we calculated colonoscopy volume as the sum of services across all colonoscopy-related CPT/HCPCS codes and providers. Providers were classified into specialty categories using NPPES taxonomy codes described earlier. We computed the proportion of colonoscopies attributed to each provider category (gastroenterology, general surgery, ambulatory surgical centers, other) as the share of total colonoscopy services contributed by that category within each census tract. Colon and rectal surgeons (taxonomy code 208C00000X) were included and grouped within the general surgery category for analysis, reflecting their role as surgical providers performing colonoscopy.

We identified ambulatory surgical centers using NPPES taxonomy code 261QA1903X, corresponding to ambulatory surgical center organizational entities; the ambulatory surgical center category therefore reflects facility-level billing rather than individual clinician specialty and may include procedures performed by multiple provider types. We summarized colonoscopy provider mix descriptively across urban and rural census tracts and in Appalachian and non-Appalachian regions, including a 4-category stratification defined by rurality/Appalachian designation. Differences in colonoscopy provider mix were assessed using distributional comparisons and visualized to highlight gradients across strata.

Next, we conducted descriptive, nonparametric, and regression analyses to characterize geographic access to colonoscopy providers and its association with CRC screening at the census tract level across the contiguous US, with a focus on Appalachian regions. Colonoscopy access was quantified using E2SFCA accessibility scores based on census tract-to-provider travel times, incorporating distance decay weights.

To compare accessibility across rural and urban tracts and Appalachian versus non-Appalachian regions, we first summarized the distribution of E2SFCA values across all tracts and examined differences by a 4-level stratification defined by rurality (urban vs rural, based on RUCA codes) and Appalachian designation. Because E2SFCA values were highly right-skewed and included zeros, we compared access across strata using the Kruskal–Wallis test. We quantified effect size, and we assessed pairwise differences with Dunn post hoc pairwise comparisons. We visualized spatial patterns of access in Appalachia using census tract–level E2SFCA quintiles.

To evaluate the association between geographic access and CRC screening prevalence, we fit beta regression models with a logit link appropriate for modeling proportional outcomes bounded between 0 and 1. Census tract–level CRC screening prevalence among adults aged 45 to 75 years was obtained from the PLACES dataset and transformed by using the Smithson–Verkuilen adjustment to accommodate boundary values. The primary exposure was log-transformed E2SFCA to improve model stability and interpretability. Models were adjusted for rural–urban status and Appalachian designation.

To account for unobserved heterogeneity in screening practices across states, we estimated mixed-effects beta regression models with a random intercept at the state level. Fixed-effect estimates are reported on the logit scale and exponentiated (Exp(β)) for interpretability, representing multiplicative effects on the odds of the mean screening proportion, with 95% CIs. We evaluated model fit and the contribution of interaction terms using likelihood ratio tests.

To assess robustness, we conducted 2 sensitivity analyses. First, we applied an alternative 4-zone distance decay specification within the E2SFCA framework, using weights of 1.00, 0.85, 0.60, and 0.40 for 0–5, >5–10, >10–30, and >30–60 minutes, respectively. Second, we recalculated E2SFCA using the population aged 65 to 74 years as the demand denominator to better align with the Medicare-based provider file. All analyses were conducted in R version 4.2.2 (R Foundation), using betareg (17), glmmTMB (18), sf (19), and data.table (20).

## Results

**Figure 1:**
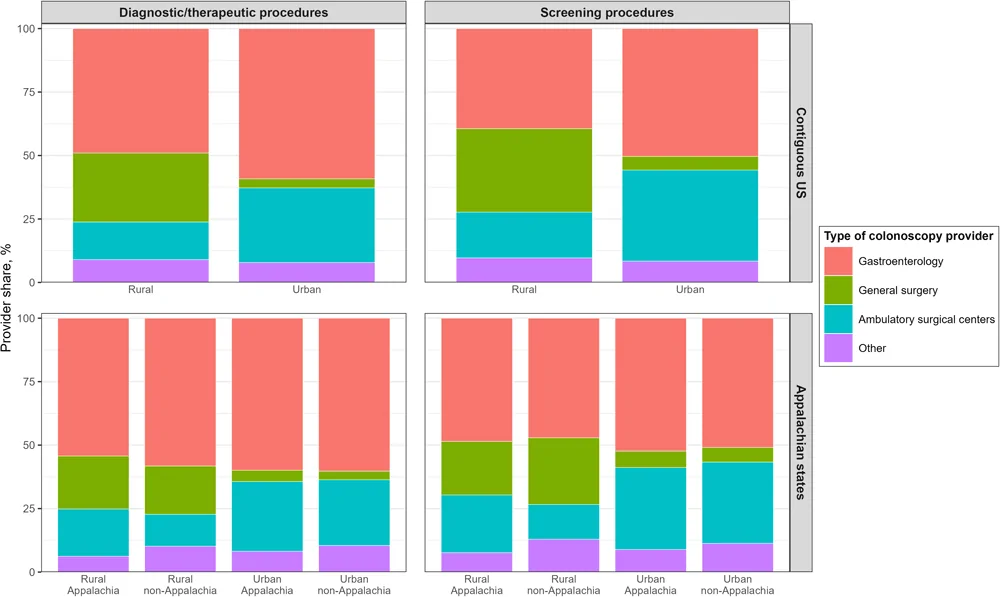
Composition of colonoscopy providers/procedures aggregated by rurality, Appalachian status, and procedure type, using 2023 Medicare Physician and Other Practitioners by Provider and Service Public Use File; CMS National Plan and Provider Enumeration System May 2025 Data Dissemination File; 2020 US Census tract boundaries; Rural–Urban Commuting Area codes 2020; and Appalachian Regional Commission county designation. The proportion of colonoscopies attributed to each provider category (gastroenterology, general surgery, ambulatory surgical centers, other) was calculated as the share of total colonoscopy services contributed by that category within each census tract.

Across the contiguous US, colonoscopy provider mix varied markedly by rurality and procedure type (Figure 1). Gastroenterologists dominated both diagnostic/therapeutic and screening procedures, but their share was consistently higher in urban areas, where ambulatory surgical centers also contributed a substantial portion of colonoscopy billing. In contrast, rural areas relied more on general surgeons, who performed only a small fraction of the colonoscopy procedures performed in urban areas but accounted for roughly one-fifth to one-quarter of colonoscopies in rural tracts. The involvement of ambulatory surgical centers showed the opposite pattern, with considerably greater representation in urban settings relative to rural ones.

**Table:** 

Geography/procedure	Stratum	Type of colonoscopy provider	No. of services provided	Provider share, %
Contiguous US				
Diagnostic/therapeutic procedures	Rural	Gastroenterology	134,041	49.0
		General surgery	74,221	27.1
		Ambulatory surgical centers	40,517	14.8
		Other	24,603	9.0
	Urban	Gastroenterology	1,704,112	59.2
		General surgery	102,479	3.6
		Ambulatory surgical centers	847,739	29.4
		Other	224,795	7.8
Screening procedures	Rural	Gastroenterology	24,854	39.4
		General surgery	20,719	32.9
		Ambulatory surgical centers	11,355	18.0
		Other	6,089	9.7
	Urban	Gastroenterology	285,329	50.4
		General surgery	30,481	5.4
		Ambulatory surgical centers	203,311	35.9
		Other	47,490	8.4
**Appalachian states**				
Diagnostic/therapeutic procedures	Rural Appalachia	Gastroenterology	28,951	54.3
		General surgery	11,112	20.8
		Ambulatory surgical centers	9,937	18.6
		Other	3,310	6.2
	Rural non-Appalachia	Gastroenterology	40,380	58.2
		General surgery	13,177	19.0
		Ambulatory surgical centers	8,737	12.6
		Other	7,044	10.2
	Urban Appalachia	Gastroenterology	100,579	59.9
		General surgery	7,454	4.4
		Ambulatory surgical centers	46,140	27.5
		Other	13,728	8.2
	Urban non-Appalachia	Gastroenterology	476,976	60.3
		General surgery	26,754	3.4
		Ambulatory surgical centers	205,077	25.9
		Other	82,684	10.4
Screening procedures	Rural Appalachia	Gastroenterology	5,807	48.6
		General surgery	2,520	21.1
		Ambulatory surgical centers	2,722	22.8
		Other	910	7.6
	Rural non-Appalachia	Gastroenterology	7,785	47.1
		General surgery	4,344	26.3
		Ambulatory surgical centers	2,258	13.7
		Other	2,136	12.9
	Urban Appalachia	Gastroenterology	17,711	52.4
		General surgery	2,174	6.4
		Ambulatory surgical centers	10,922	32.3
		Other	3,011	8.9
	Urban non-Appalachia	Gastroenterology	79,027	50.9
		General surgery	8,965	5.8
		Ambulatory surgical centers	49,643	32.0
		Other	17,575	11.3

These gradients persisted in Appalachian states. Urban Appalachian census tracts resembled other urban regions, with high gastroenterology and ambulatory surgery center involvement and minimal general surgery involvement. Rural Appalachian census tracts shifted toward greater reliance on general surgery and a reduced reliance on ambulatory surgery centers, although gastroenterology remained the majority billing group across all strata. The 4-category stratification highlighted these differences more sharply: ambulatory surgery center billing consistently peaked in urban strata, whereas general surgery billing was highest in both rural strata, particularly in rural Appalachia. Rural tracts demonstrated substantially greater reliance on general surgeons than urban areas, indicating a shift in colonoscopy delivery from gastroenterologists toward general surgeons.

Across census tracts, the E2SFCA colonoscopy access index exhibited a relatively narrow distribution, indicating limited variation in geographic access across most areas. On the log scale, the difference between the 10th and 90th percentiles was 0.84.

**Figure 2:**
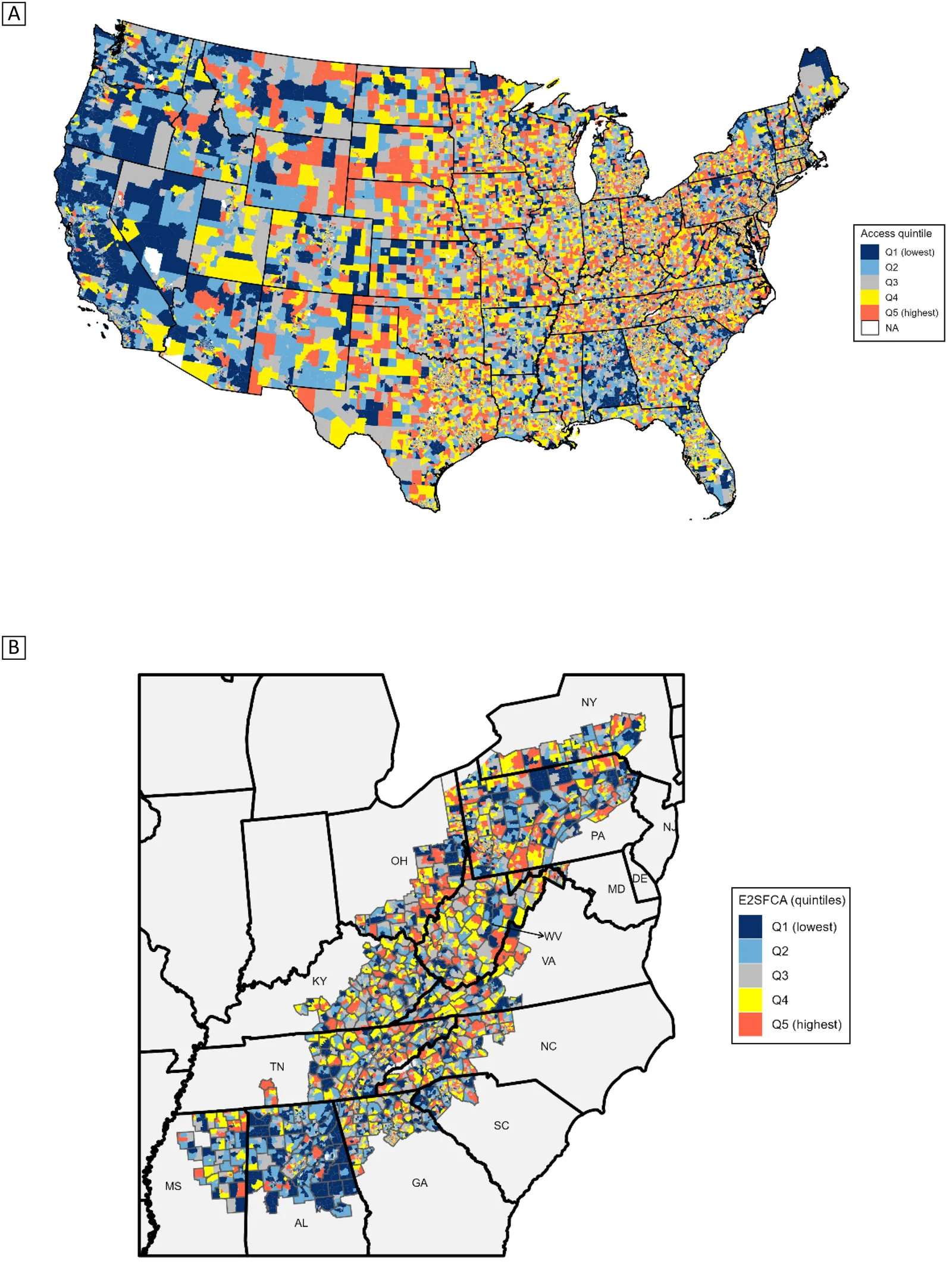
Geographic access to colonoscopy providers at the census tract level in A) the contiguous US and B) in Appalachian states, 2020 US Census tract boundaries and American Community Survey 2019–2023 5-year estimates. Access quintiles (Map A) and E2SFCA quintiles (Map B) represent the same E2SFCA accessibility measure, categorized into quintiles, with Q1 indicating the lowest geographic access and Q5 indicating the highest geographic access. Panel B provides a zoomed-in view of the Appalachian region. Abbreviations: E2SFCA, enhanced 2-step floating catchment area; NA, not applicable

Additionally, although nonparametric tests detected significant differences in E2SFCA across rural/Appalachia strata (Kruskal–Wallis χ^2^_3_ = 692.4, *P* < .001), the magnitude of separation was negligible in practical terms (ε^2^ = 0.0083). Dunn post hoc tests showed that all pairwise comparisons were significant. Across comparisons, effect sizes were uniformly small (*r* = 0.008–0.076), indicating minimal practical separation between strata. We found substantial spatial heterogeneity in census tract–level geographic access to colonoscopy providers across Appalachia, as measured by E2SFCA quintiles (Figure 2). Higher-access tracts (Q4–Q5) tended to cluster around urban centers and regional medical hubs, while large contiguous areas of central and southern Appalachia were characterized by lower access (Q1–Q2). The small-area variation within states and counties highlights that access is highly localized and influenced by colonoscopy provider location, travel constraints, and population distribution.

In the evaluation of the association between census tract–level CRC screening and geographic access to colonoscopy providers, after adjusting for rural–urban status, Appalachian designation, and state-level variation, colonoscopy access (log10(E2SFCA)) was not significantly associated with CRC screening prevalence (Exp(β) = 1.000; 95% CI, 0.994–1.006; *P* = .95) (Table).

**Table:** Beta Regression Model: Access and Colorectal Cancer Screening Prevalence, Using CDC PLACES 2023 Release, 2020 US Census Tract Boundaries, American Community Survey 2019–2023 5-Year Estimates, Rural–Urban Commuting Area Codes, Appalachian Regional Commission Designation, and Derived E2SFCA Accessibility Measures

Predictor	Estimate (β)	Exp(β)	SE	95% CI	*P* value
**Fixed effects**					
Intercept	0.6405	1.897	0.0251	1.806–1.993	<.001
Colonoscopy accessibility (log10(E2SFCA))	−0.0002	1.000	0.0031	0.994–1.006	.95
Rural (vs urban)	−0.0060	0.994	0.0027	0.989–0.999	.02
Appalachian (vs non-Appalachian)	−0.0247	0.976	0.0043	0.967–0.984	<.001
**Random effects**					
State-level intercept variance	0.0206	—	—	—	—
State-level intercept SD	0.1437	—	—	—	—

Abbreviations: CDC, Centers for Disease Control and Prevention; E2SFCA, enhanced 2-step floating catchment area; —, does not apply.

Rural tracts had slightly lower CRC screening prevalence compared with urban tracts (Exp(β) = 0.994; 95% CI, 0.989–0.999; *P* = .02). Appalachian tracts also had lower CRC screening prevalence than non-Appalachian tracts (Exp(β) = 0.976; 95% CI, 0.967–0.984; *P* < .001). The state-level random intercept standard deviation (0.1437) indicates modest between-state heterogeneity; a 1–SD higher state intercept corresponds to approximately a 15% increase on the exponentiated scale (exp[0.1437] = 1.16), conditional on covariates.

In the first sensitivity analysis, in which we applied an alternative distance decay specification, colonoscopy access remained not significantly associated with CRC screening prevalence (Exp(β) = 0.996; 95% CI: 0.990–1.002; *P* = .24). Appalachian designation remained associated with lower screening prevalence, while the rural–urban difference was attenuated and not significant. In the second sensitivity analysis, which used the population aged 65 to 74 as the demand denominator, the absolute scale of the accessibility index was changed, but the spatial pattern of access was preserved; the age-matched and primary accessibility measures were highly correlated on both the original scale (*r* = 0.999) and log scale (*r* = 0.999). Accordingly, regression results were largely unchanged, with colonoscopy access remaining not significantly associated with CRC screening prevalence. Rural status was no longer significant in this sensitivity model, although the estimate remained small and in the same direction; Appalachian designation remained significantly associated with lower CRC screening prevalence.

## Discussion

This national analysis demonstrated substantial geographic and structural heterogeneity in the US colonoscopy delivery system, with clear rural–urban and Appalachian–non-Appalachian gradients. Consistent with prior work documenting limited specialty presence in rural settings (21,22), we found that general surgeons provide a disproportionately large share of colonoscopy services in rural counties, particularly in rural Appalachia. In contrast, urban areas both in and outside Appalachia were characterized by gastroenterologist-dominated delivery supplemented by high ambulatory surgery center presence, mirroring established patterns of endoscopy concentration in urban and suburban markets (23,24). These findings underscore the persistent patterns of specialty care and the reliance on general surgeons to maintain procedural capacity in rural health systems. This pattern suggests that efforts to improve access in these areas may benefit from incorporating general surgeons into workforce planning. Prior work has noted that rural general surgeons often provide screening services (21) but may face structural and funding constraints.

Geographic access varied modestly across rural–urban and Appalachian strata. Although nonparametric tests detected significant differences in E2SFCA distributions, overall access levels were tightly clustered, with minimal practical separation between groups. Median E2SFCA values were comparable across all 4 strata, and access to colonoscopy in rural Appalachia was not systematically lower than in other regions. These findings suggest that, when measured by using travel-time–weighted provider availability, potential geographic access to colonoscopy services is relatively uniform across strata, despite well-documented differences in provider mix and health system structure (25–28).

The magnitude of disparity was modest. After adjusting for rural–urban status and state-level variation, Appalachian census tracts had about 2.4% lower relative CRC screening prevalence than non-Appalachian census tracts (Exp(β)=0.976; 95% CI, 0.0967–0.984; *P* < .001). This pattern was robust to alternative distance decay specifications, although the rural–urban difference was attenuated and no longer significant in sensitivity analyses, while Appalachian disparities remained consistent.

The association between E2SFCA-based geographic access and tract-level CRC screening prevalence was not significant. These findings indicate that potential geographic access to colonoscopy providers by itself does not explain differences in community-level screening uptake, consistent with evidence that spatial access is only 1 component of multilevel barriers to CRC screening (29–31). Multiple nongeographic barriers, including health insurance coverage, out-of-pocket costs, health literacy, mistrust, competing demands, and primary care referral practices play substantial roles in shaping CRC screening behavior (32–34).

Although geographic access was not significantly associated with CRC screening in the regression models, urban Appalachian census tracts showed a provider mix more similar to other urban areas (higher gastroenterologist and ambulatory surgery center involvement) than to rural strata. Differences in care delivery organization and enabling factors beyond geographic access may contribute to these patterns (23). Conversely, lack of association between access and screening suggests that spatial proximity alone does not meaningfully explain differences in CRC screening, including in rural and Appalachian communities. This finding is consistent with prior E2SFCA studies of endoscopy and oncology care, which similarly reported weak or null associations between geographic access measures and screening utilization or stage at diagnosis (35).

### Limitations

Interpretation of patterns of provider mix must consider several data constraints. First, CMS specialty codes identify the billing provider rather than the physical endoscopy setting; thus, procedures billed by gastroenterologists may have been performed in ambulatory surgical centers, and ambulatory surgical center claims may include services delivered by multiple clinician types. This limited our ability to characterize the true procedural environment. Second, Medicare Part B claims capture only reimbursed services and therefore exclude colonoscopies paid by Medicaid, commercial insurance, out-of-pocket arrangements, and hospital outpatient departments using alternative billing mechanisms. These omissions may disproportionately affect rural and Appalachian regions and may underrepresent certain provider groups.

Third, the grouping of clinician types (gastroenterology, general surgery, ambulatory surgical center, other) is constrained by Medicare specialty codes and may obscure heterogeneity within each category, including advanced practice proceduralists and subspecialty surgeons. Fourth, billing-based procedure counts reflect service provision rather than clinical appropriateness, indication, or patient-level characteristics; thus, observed patterns cannot distinguish differences in supply, referral pathways, or population need. Fifth, aggregation to rural–urban and Appalachian strata describes population-level patterns and masks within-stratum variability.

Limitations also apply to the access and screening analyses. Because the E2SFCA index represents potential geographic access rather than realized utilization, it does not capture data on wait times, scheduling constraints, or endoscopy capacity. PLACES screening estimates are modeled small-area predictions based on Behavioral Risk Factor Surveillance System data and census/American Community Survey data and do not represent direct measurement. Therefore, associations between E2SFCA and screening should be interpreted as ecological correlations conditioned on the structure of the PLACES modeling framework. Furthermore, interpretation of log-scale pairwise differences reflects multiplicative, not additive, contrasts; the derived ratios describe relative differences in central tendency and do not represent arithmetic mean differences on the original E2SFCA scale.

Finally, geographic access to colonoscopy providers was measured at the census tract level using residential (“nighttime”) population locations, which may not reflect actual care-seeking behavior. Patients, particularly in rural areas, may obtain services near workplaces or regional referral centers rather than near their place of residence. In addition, census tracts can be geographically large and internally heterogeneous, potentially obscuring within-tract variation in access. Travel time estimates based on census tract centroids may further introduce measurement error, because centroids may not correspond to true population locations or road network access points.

### Conclusion

This national study found structural differences in the colonoscopy provider landscape across rural and Appalachian communities. Provider mix varied systematically by rurality, with urban tracts relying predominantly on gastroenterologists and ambulatory surgical centers, whereas rural areas, particularly rural Appalachia, depended more on general surgeons for both screening and diagnostic /therapeutic colonoscopy services (29).

Geographic access measured by using a travel-time–weighted E2SFCA index showed limited variation across strata and was not associated with CRC screening prevalence. Appalachian disparities persisted after adjustment for access and state-level variation, while rural differences were small and not robust to sensitivity analyses (36), indicating that factors beyond geographic proximity, such as health care organization, socioeconomic conditions, and utilization barriers, likely play a more substantial role in shaping screening behavior.
